# Supplementary figures and images for: Mechanistic model for human brain metabolism and its connection to the neurovascular coupling
Source: PLoS Comput Biol. 2022 Dec 22;18(12):e1010798. doi: 10.1371/journal.pcbi.1010798 (PMC9822108; doi:10.1371/journal.pcbi.1010798)

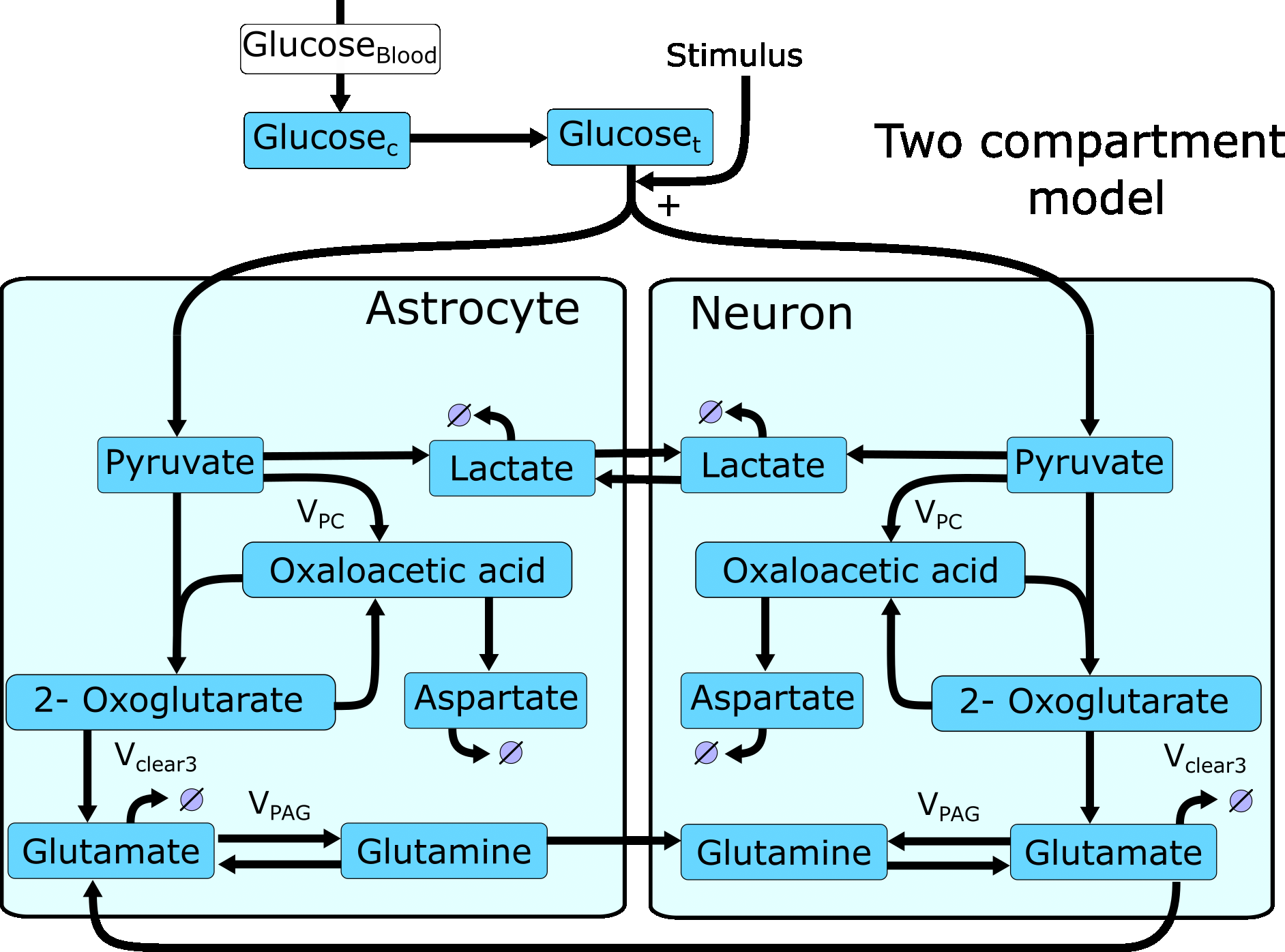

Supplement: S1 Fig — Illustrating the division of the metabolic reactions into a glial and a neutron compartment with some cross over reactions. (TIF) [file pcbi.1010798.s001.tif]

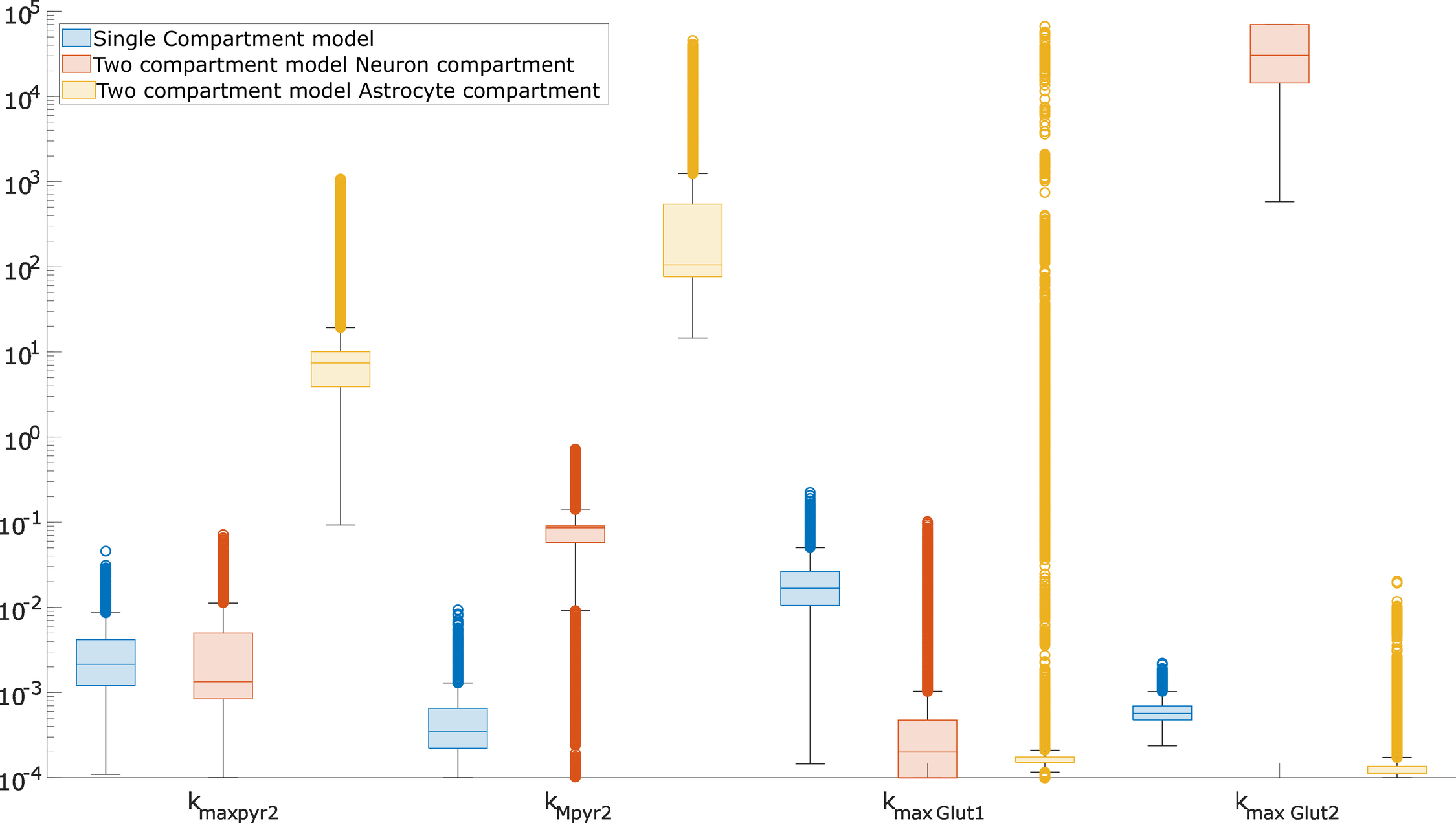

Supplement: S2 Fig — The box chart illustrates the parameter distributions for four parameters (kmaxpyr2, kMPyr2, KmaxGlut1, kmaxGlut2). With the distribution of the parameters in the single compartment model shown in blue and distributions of the corresponding parameters for the two-compartment model shown in red and yellow respectively. (TIF) [file pcbi.1010798.s002.tif]
