# Supplementary material for: Mechanistic model for human brain metabolism and its connection to the neurovascular coupling
Source: PLoS Comput Biol. 2022 Dec 22;18(12):e1010798. doi: 10.1371/journal.pcbi.1010798 (PMC9822108; doi:10.1371/journal.pcbi.1010798)
Supplement: S1 Table — 2017 model [23]. The equations are taken with the consent of the authors of the original work and are presented here as for the convenience of the reader. Please see the original publication for a detailed description of the model equations. (DOCX) [file pcbi.1010798.s003.docx]

# S1 Table: Implementation of Sten *et al.* 2017 [1] model

| **State Equations** | **Interpretation** |
| --- | --- |
| $\frac{d(Stimulus)}{dt} = 0$ | Stimulus input signal |
| $\frac{d(oHb)}{dt}= {v1}_{b}- {v1}_{f}+v_{inoHb}-v_{outoHb}$ | Change in oxyhemoglobin level |
| $\frac{d(dHb)}{dt}={v1}_{f}- {v1}_{b}+v_{indHb}-v_{outdHb}$ | Change in deoxyhemoglobin level |
| $\frac{d\left( O_{2A} \right)}{dt}=v_{O_{2A}} \times v_{bv}-v_{basalMet} \times k_{prop1}- v_{stimMet} \times k_{prop2}*k_{volumeScale2}$ | Change in free oxygen in blood compartment |
| $\frac{d\left( O_{2B} \right)}{dt}={v1}_{f}- {v1}_{b}-v_{O_{2A}}+v_{inO_{2}}-v_{outO_{2}}$ | Change in free oxygen in extravascular tissue |
| $\frac{d\left( {Glucose}_{A} \right)}{dt}={(v}_{Gluc1}+ v_{Gluc2})v_{bv}*k_{volumeScale}-v_{basalMet}- v_{stimMet}$ | Change in glucose (blood compartment) |
| $\frac{d\left( {Glucose}_{B} \right)}{dt}=-\left( v_{Gluc1}+ v_{Gluc2} \right)+v_{inG}-v_{outG}$ | Change in glucose level in extravascular tissue |
| $\frac{d\left( {Delay}_{M} \right)}{dt}= {input}_{1}-{met}_{sink}$ | Delay state |
| $\frac{d(Glutamate)}{dt} = {input}_{2} - {Glutamate}_{sink}$ | Glutamate release upon stimulation |
| $\frac{d(GABA)}{dt} = {input}_{3} - {GABA}_{sink}$ | GABA release upon stimulation |
| $\frac{d\left( {Ca}^{2+} \right)}{dt} = k_{Ca}\left( 1+v_{glutamate} \right)$  $\left( \frac{1}{1+v_{GABA}\left( 1+v_{diazepam} \right)} \right)-{Ca}_{sink}$ | Calcium influx in the astrocyte |
| $\frac{d(AA)}{dt} = v_{calcium}-\left( k_{vc}+k_{vd} \right)AA$ | Change in AA level |
| $\frac{d(\text{AA-met}_{\text{vc1}})}{dt} = k_{vc}AA-v_{vc1}$  $\frac{d(\text{AA-met}_{\text{vc2}})}{dt} = v_{vc1}-v_{vc2}$  $\frac{d(\text{AA-met}_{\text{vc3}})}{dt} = v_{vc2}-v_{vc3}$  $\frac{d(\text{AA-met}_{\text{vc4}})}{dt} = v_{vc3}-v_{vc4}$ | Intermediary states |
| $\frac{d(\text{AA-met}_{\text{vd1}})}{dt} = k_{vd}AA-v_{vd1}$  $\frac{d(\text{AA-met}_{\text{vd2}})}{dt} = v_{vd1}-v_{vd2}$  $\frac{d(\text{AA-met}_{\text{vd3}})}{dt} = v_{vd2}-v_{vd3}$  $\frac{d(\text{AA-met}_{\text{vd4}})}{dt} = v_{vd3}-v_{vd4}$ | Intermediary states |
| **Reactions** | **Interpretation** |
| ${v1}_{f} = {k1}_{f} \times oHb$  ${v1}_{b} = {k1}_{b} \times dHb \times O_{2B}$ | Rate of releasing oxyhemoglobin into oxygen and deoxyhemoglobin  Rate of binding oxygen and deoxyhemoglobin into oxyhemoglobin |
| $v_{inoHb} = {oHb}_{basal} \times v_{flow}$  $v_{outoHb} = oHb \times v_{flow}$  $v_{indHb} = {dHb}_{basal} \times v_{flow}$  $v_{outdHb} = dHb \times v_{flow}$  $v_{inG} = {{(Glucose}_{B})}_{basal} \times v_{flow}$  $v_{outG} = {Glucose}_{B} \times v_{flow}$  $v_{inO_{2}} = {{(O}_{2B})}_{basal} \times v_{flow}$  $v_{outO_{2}} = O_{2B} \times v_{flow}$ | Oxyhemoglobin influx  Oxyhemoglobin outflux  Deoxyhemoglobin influx  Deoxyhemoglobin outflux  Glucose influx  Glucose outflux  Oxygen influx  Oxygen outflux |
| $v_{O_{2A}}=k_{O2}\left( O_{2B}-O_{2A} \right)$  $v_{Gluc1}=k_{Gluc1}\left( {Glucose}_{B}-{Glucose}_{A} \right)$  $v_{Gluc2}=k_{Gluc2}\left( \frac{{Glucose}_{B}}{k_{m}+{Glucose}_{B}} \right)$ | Diffusion of oxygen between blood and cell compartment  Diffusion of glucose between blood and cell compartment  Receptor mediated transportation of glucose between blood and cell compartment |
| $v_{basalMet} = k_{basalMet} \times{O_{2A}}^{prop1} \times{Glucose}_{A}$  $v_{stimMet} = {Delay}_{M} \times{O_{2A}}^{prop2} \times{Glucose}_{A}$ | Basal metabolism  Stimulation induced metabolism |
| ${input}_{1} = k_{met} \times Stimulus$  ${input}_{2} = k_{Glutamate}\times Stimulus$  ${input}_{3} = k_{GABA}\times Stimulus$  ${Glutamate}_{Sink} = {sink}_{Glutamate} \times Glutamate$  ${GABA}_{Sink} = {sink}_{GABA} \times GABA$  ${met}_{Sink} ={sink}_{met} \times{Delay}_{M}$ | Stimulus input to the metabolic module  Stimulus input to glutamate release  Stimulus input to GABA release  Glutamate degradation  GABA degradation  Degradation of increased metabolism |
| $v_{glutamate} = k_{3} \times Glutamate$  $v_{GABA} = \frac{GABA}{k_{4}}$  $v_{diazepam} = \frac{c_{diaz}^{n}}{k_{diaz}^{n}+c_{diaz}^{n}}$  ${Ca}_{sink} = {sink}_{Ca} \times{Ca}^{2+}$  $v_{calcium}= k_{PL} \times{Ca}^{2+}$ | Glutamate’s effect on calcium  GABA’s effect on calcium  Diazepam’s effect on calcium  Degradation of calcium  Calcium’s effect on AA |
| $v_{vc1} = k_{vc1} \times\text{AA-met}_{\text{vc1}}$  $v_{vc2} = k_{vc2} \times\text{ AA-met}_{\text{vc2}}$  $v_{vc3} = k_{vc3} \times\text{ AA-met}_{\text{vc3}}$  $v_{vc4} =k_{vc4} \times\text{ AA-met}_{\text{vc4}}$ | Delay states |
| $v_{vd1} = k_{vd1} \times\text{AA-met}_{\text{vd1}}$  $v_{vd2} = k_{vd2} \times\text{ AA-met}_{\text{vd2}}$  $v_{vd3} = k_{vd3} \times\text{ AA-met}_{\text{vd3}}$  $v_{vd4} =k_{vd4} \times\text{ AA-met}_{\text{vd4}}$ | Delay states |
| **Variable values** | **Interpretation** |
| ${v_{flow}=k}_{flow} + e^{k_{s} \times\text{AA-met}_{\text{vd4}} - {k_{i} \times\text{AA-met}}_{\text{vc4}}}$ | Blood flow |
| ${v_{bv}=\left( v_{flow} \right)}^{k_{bv}}$ | Blood volume |
| $Glucos{e_{B}}_{basal}=$10 | Basal amount of glucose in blood |
| $oHb_{basal}=$10 | Basal amount of oxygen in blood |
| ${O_{2B}}_{basal}=$10 | Basal amount of oxygenated hemoglobin in blood |
| $dHb_{basal}=$10 | Basal amount of deoxygenated hemoglobin in blood |
| $BOLD=e^{-k_{y} \times dHb}$ | Measurement signal |

# References

1. Sten S, Lundengård K, Witt ST, Cedersund G, Elinder F, Engström M. Neural inhibition can explain negative BOLD responses: A mechanistic modelling and fMRI study. NeuroImage. 2017 Sep 1;158:219–31.
