# Supplementary material for: Mechanistic model for human brain metabolism and its connection to the neurovascular coupling
Source: PLoS Comput Biol. 2022 Dec 22;18(12):e1010798. doi: 10.1371/journal.pcbi.1010798 (PMC9822108; doi:10.1371/journal.pcbi.1010798)
Supplement: S2 Table — The calculations are based on an implementation of the methods presented by Thompson et al., 2022 [40]. (DOCX) [file pcbi.1010798.s004.docx]

# S2 Table: Structural identifiability Analysis

We performed a local identifiability analysis on the metabolism model described in section 2.3.1. We implemented recently developed methods described by Thompson *et al.*, 2022 [1], which determine local identifiability of states and parameters when different numbers of derivatives of signals are available.

Since the four steady state values of the signals were known, we substituted these numerical values for the parameters: ssLac = 0.00014149267643708, ssGlut = 4.14433242733474e-06, ssGluct = 636216.973627674, ssAsp = 0.0284289654822431.

To accommodate the use of a locally constant input u, we replaced u with 1 in the model equations. All states but Stimulus and all parameters but kstim1 and kmaxgluct are identifiable if at least seven derivatives of the signals are available.

Local identifiability of states and parameters in metabolism model with u replaced by 1

| numax | 0 | 1 | 2 | 3 | 4 | 5 | 6 | 7 | 8 | 9 | … | 30 | 31 | 32 |
| --- | --- | --- | --- | --- | --- | --- | --- | --- | --- | --- | --- | --- | --- | --- |
| Stimulus | no | no | no | no | no | no | no | no | no | no | … | no | no | no |
| Gluct | no | no | no | no | no | no | no | yes | yes | yes | … | yes | yes | yes |
| Glucc | no | no | no | no | no | no | no | yes | yes | yes | … | yes | yes | yes |
| Pyr | no | no | no | no | no | no | no | yes | yes | yes | … | yes | yes | yes |
| Lac | no | no | no | no | no | no | no | yes | yes | yes | … | yes | yes | yes |
| OAA | no | no | no | no | no | no | no | yes | yes | yes | … | yes | yes | yes |
| OG | no | no | no | no | no | no | no | yes | yes | yes | … | yes | yes | yes |
| Asp | no | no | no | no | no | no | no | yes | yes | yes | … | yes | yes | yes |
| Glut | no | no | no | no | no | no | no | yes | yes | yes | … | yes | yes | yes |
| Gln | no | no | no | no | no | no | no | yes | yes | yes | … | yes | yes | yes |
| kstim1 | no | no | no | no | no | no | no | no | no | no | … | no | no | no |
| kstim2 | no | no | no | no | no | no | no | yes | yes | yes | … | yes | yes | yes |
| kmaxglucc | no | no | no | no | no | no | no | yes | yes | yes | … | yes | yes | yes |
| GlucosBlood | no | no | no | no | no | no | no | yes | yes | yes | … | yes | yes | yes |
| kmaxgluct | no | no | no | no | no | no | no | no | no | no | … | no | no | no |
| kmaxPO | no | no | no | no | no | no | no | yes | yes | yes | … | yes | yes | yes |
| KMpyr | no | no | no | no | no | no | no | yes | yes | yes | … | yes | yes | yes |
| KMOAA | no | no | no | no | no | no | no | yes | yes | yes | … | yes | yes | yes |
| kmaxPyr | no | no | no | no | no | no | no | yes | yes | yes | … | yes | yes | yes |
| kmaxPyr2 | no | no | no | no | no | no | no | yes | yes | yes | … | yes | yes | yes |
| KMPyr2 | no | no | no | no | no | no | no | yes | yes | yes | … | yes | yes | yes |
| k1 | no | no | no | no | no | no | no | yes | yes | yes | … | yes | yes | yes |
| kmaxOG1 | no | no | no | no | no | no | no | yes | yes | yes | … | yes | yes | yes |
| kmaxOAA | no | no | no | no | no | no | no | yes | yes | yes | … | yes | yes | yes |
| kmaxOG2 | no | no | no | no | no | no | no | yes | yes | yes | … | yes | yes | yes |
| kmaxAsp | no | no | no | no | no | no | no | yes | yes | yes | … | yes | yes | yes |
| kmaxGln | no | no | no | no | no | no | no | yes | yes | yes | … | yes | yes | yes |
| kmaxGlut1 | no | no | no | no | no | no | no | yes | yes | yes | … | yes | yes | yes |
| kmaxGlut2 | no | no | no | no | no | no | no | yes | yes | yes | … | yes | yes | yes |
| ky1 | no | no | no | no | no | no | no | yes | yes | yes | … | yes | yes | yes |
| ky2 | no | no | no | no | no | no | no | yes | yes | yes | … | yes | yes | yes |
| ky3 | no | no | no | no | no | no | no | yes | yes | yes | … | yes | yes | yes |
| ky4 | no | no | no | no | no | no | no | yes | yes | yes | … | yes | yes | yes |

To describe the possibility of using a different input signal for u, we performed the analysis on the model equations as written in section 2.3.1. All states and parameters are identifiable if at least eight derivatives of the signals are available.

Local identifiability of states and parameters in metabolism model with arbitrary u

| numax | 0 | 1 | 2 | 3 | 4 | 5 | 6 | 7 | 8 | 9 | 10 | … | 30 | 31 | 32 |
| --- | --- | --- | --- | --- | --- | --- | --- | --- | --- | --- | --- | --- | --- | --- | --- |
| Stimulus | no | no | no | no | no | no | no | no | yes | yes | yes | … | yes | yes | yes |
| Gluct | no | no | no | no | no | no | no | no | yes | yes | yes | … | yes | yes | yes |
| Glucc | no | no | no | no | no | no | no | no | yes | yes | yes | … | yes | yes | yes |
| Pyr | no | no | no | no | no | no | no | no | yes | yes | yes | … | yes | yes | yes |
| Lac | no | no | no | no | no | no | no | no | yes | yes | yes | … | yes | yes | yes |
| OAA | no | no | no | no | no | no | no | no | yes | yes | yes | … | yes | yes | yes |
| OG | no | no | no | no | no | no | no | no | yes | yes | yes | … | yes | yes | yes |
| Asp | no | no | no | no | no | no | no | no | yes | yes | yes | … | yes | yes | yes |
| Glut | no | no | no | no | no | no | no | no | yes | yes | yes | … | yes | yes | yes |
| Gln | no | no | no | no | no | no | no | no | yes | yes | yes | … | yes | yes | yes |
| kstim1 | no | no | no | no | no | no | no | no | yes | yes | yes | … | yes | yes | yes |
| kstim2 | no | no | no | no | no | no | no | no | yes | yes | yes | … | yes | yes | yes |
| kmaxglucc | no | no | no | no | no | no | no | no | yes | yes | yes | … | yes | yes | yes |
| GlucosBlood | no | no | no | no | no | no | no | no | yes | yes | yes | … | yes | yes | yes |
| kmaxgluct | no | no | no | no | no | no | no | no | yes | yes | yes | … | yes | yes | yes |
| kmaxPO | no | no | no | no | no | no | no | no | yes | yes | yes | … | yes | yes | yes |
| KMpyr | no | no | no | no | no | no | no | no | yes | yes | yes | … | yes | yes | yes |
| KMOAA | no | no | no | no | no | no | no | no | yes | yes | yes | … | yes | yes | yes |
| kmaxPyr | no | no | no | no | no | no | no | no | yes | yes | yes | … | yes | yes | yes |
| kmaxPyr2 | no | no | no | no | no | no | no | no | yes | yes | yes | … | yes | yes | yes |
| KMPyr2 | no | no | no | no | no | no | no | no | yes | yes | yes | … | yes | yes | yes |
| k1 | no | no | no | no | no | no | no | no | yes | yes | yes | … | yes | yes | yes |
| kmaxOG1 | no | no | no | no | no | no | no | no | yes | yes | yes | … | yes | yes | yes |
| kmaxOAA | no | no | no | no | no | no | no | no | yes | yes | yes | … | yes | yes | yes |
| kmaxOG2 | no | no | no | no | no | no | no | no | yes | yes | yes | … | yes | yes | yes |
| kmaxAsp | no | no | no | no | no | no | no | no | yes | yes | yes | … | yes | yes | yes |
| kmaxGln | no | no | no | no | no | no | no | no | yes | yes | yes | … | yes | yes | yes |
| kmaxGlut1 | no | no | no | no | no | no | no | no | yes | yes | yes | … | yes | yes | yes |
| kmaxGlut2 | no | no | no | no | no | no | no | no | yes | yes | yes | … | yes | yes | yes |
| ky1 | no | no | no | no | no | no | no | no | yes | yes | yes | … | yes | yes | yes |
| ky2 | no | no | no | no | no | no | no | no | yes | yes | yes | … | yes | yes | yes |
| ky3 | no | no | no | no | no | no | no | no | yes | yes | yes | … | yes | yes | yes |
| ky4 | no | no | no | no | no | no | no | no | yes | yes | yes | … | yes | yes | yes |

In both tables, numax is the number of derivatives of all signals assumed to be available. For example, if numax = 3, then the 0^th^, 1^st^, 2^nd^, and 3^rd^ derivatives of the four signals are assumed available and the 4^th^, 5^th^ etc. unavailable.

# References

1. Thompson P, Andersson BJ, Cedersund G. A new method for a priori practical identifiability. bioRxiv; 2022. p. 2022.10.20.511900. doi: 10.1101/2022.10.20.511900
