# Supplementary material for: Mechanistic model for human brain metabolism and its connection to the neurovascular coupling
Source: PLoS Comput Biol. 2022 Dec 22;18(12):e1010798. doi: 10.1371/journal.pcbi.1010798 (PMC9822108; doi:10.1371/journal.pcbi.1010798)
Supplement: S3 Table — The parameter values correspond to the values obtained for the best fit to the estimation data. The upper and lower bounds correspond to the range of acceptable parameter values obtained from the uncertainty analysis. (DOCX) [file pcbi.1010798.s005.docx]

# S3 Table: Parameter values

| Parameter Name | Unit | Optimal Parameter Value | Upper parameter bound | Lower parameter bound |
| --- | --- | --- | --- | --- |
| ky | unitless | 7.3000 | 56.820 | 0.0100 |
| kmetabolic | 1/s | 0.0002 | 0.3200 | 0.0001 |
| k1f | 1/(amount$\times$s) | 0.0003 | 0.0810 | 0.0001 |
| k1b | 1/(amount$\times$s) | 0.1300 | 3.1100 | 0.0010 |
| $k_{basalMet}$ | 1/s | 0.0500 | 0.0070 | 0.0010 |
| kflowBasal | amount/s | 0.0600 | 0.8900 | 0.0001 |
| $k_{prop1}$ | Ratio of oxygen/glucose metabolised | 0.1200 | 1.1000 | 0.0001 |
| $k_{prop2}$ | Ratio of oxygen/glucose metabolized | 0.0100 | 0.3500 | 0.0001 |
| kO2bbb | 1/s | 0.0600 | 20.670 | 0.0001 |
| kGlucf | 1/s | 0.9000 | 22.760 | 0.0020 |
| kGlucb | 1/s | 0.0001 | 0.0100 | 0.0001 |
| kBV | unitless | 1.6000 | 96.820 | 0.0001 |
| Kmgluc | amount | 0.0100 | 10.240 | 0.0001 |
| kGD | 1/s | 0.0600 | 95.270 | 0.0200 |
| PL | 1/s | 0.1200 | 69911.0 | 0.0001 |
| CaBas | 1/s | 0.0200 | 32615.0 | 0.0001 |
| sinkGABA | 1/s | 0.2500 | 2.3200 | 0.0001 |
| sinkGlu | 1/s | 1.0200 | 5.3900 | 0.2400 |
| sinkA | 1/s | 1.2300 | 7.2300 | 0.3400 |
| sinkCon | 1/s | 0.8800 | 9.5100 | 0.3000 |
| sinkDil | 1/s | 0.6400 | 69990.0 | 0.2400 |
| Kglut | 1/s | 0.1300 | 0.3200 | 0.0010 |
| Kgaba | 1/s | 0.0001 | 0.4700 | 0.0001 |
| k3 | 1/s | 886.93 | 69999.0 | 427.37 |
| k4 | amount$\times$s | 26.710 | 26222.0 | 0.0002 |
| k5 | 1/s | 1.0100 | 7.3000 | 0.2800 |
| k7 | 1/s | 0.1100 | 1.0500 | 0.0001 |
| b3 | unitless | 1.1800 | 69994.0 | 0.0100 |
| b4 | unitless | 0.2200 | 214.41 | 0.0001 |
| kdelay1d | 1/s | 0.4400 | 4287.7 | 0.1400 |
| kdelay2d | 1/s | 55521.0 | 69999.0 | 0.5400 |
| kdelay3d | 1/s | 2.5400 | 59.820 | 0.1300 |
| kdelay1c | 1/s | 1.2100 | 7.2800 | 0.3600 |
| kdelay2c | 1/s | 0.9100 | 10.760 | 0.3900 |
| kdelay3c | 1/s | 1.4900 | 108.09 | 0.4600 |
| ${k_{max}}_{pyr}$ | 1/s | 61.230 | 7069.9 | 0.0100 |
| ${K_{M}}_{pyr}$ | amount | 0.7700 | 69266.0 | 0.0030 |
| k1 | 1/s | 0.0050 | 0.0100 | 0.0020 |
| ky1 | Unitless | 0.0001 | 42201.0 | 0.0001 |
| ky2 | Unitless | 69890.0 | 69999.0 | 0.0800 |
| ${k_{max}}_{OAA}$ | 1/s | 0.0007 | 0.0100 | 0.0001 |
| ${K_{M}}_{OAA}$ | amount | 0.0001 | 0.1500 | 0.0001 |
| ${k_{max}}_{OG1}$ | 1/s | 0.1900 | 69964.0 | 0.0100 |
| ${k_{max}}_{OG2}$ | 1/s | 0.0200 | 31384.0 | 0.0004 |
| ${k_{max}}_{Glut1}$ | 1/s | 0.0300 | 2.9100 | 0.0001 |
| ${k_{max}}_{Glut2}$ | 1/s | 0.0010 | 0.0040 | 0.0001 |
| ${k_{max}}_{Gln}$ | 1/s | 0.0200 | 2.1800 | 0.0001 |
| ${k_{max}}_{Asp}$ | 1/s | 0.0300 | 69900.0 | 0.0004 |
| ${k_{max}}_{PO}$ | 1/s | 14.290 | 69980.0 | 0.0100 |
| ky3 | Unitless | 1993.3 | 69854.0 | 0.0001 |
| ky4 | Unitless | 46.830 | 69990.0 | 0.0100 |
| ${k_{max}}_{Pyr2}$ | 1/s | 0.0200 | 0.2600 | 0.0001 |
| $k_{stim1}$ | Unitless | 0.0060 | 69981.0 | 0.0001 |
| $k_{stim2}$ | Unitless | 2588.0 | 69830.0 | 0.0001 |
| ${K_{M}}_{Pyr2}$ | Amount | 0.0020 | 0.4400 | 0.0001 |
| k_volumeScale | Unitless | 0.4900 | 8.5900 | 0.0002 |
| k_volumeScale2 | Unitless | 5.8600 | 788.74 | 0.0001 |
| Neg_kglut | 1/s | 0.1800 | 55584.0 | 0.0010 |
| Neg_kgaba | 1/s | 714.54 | 69999.0 | 0.0400 |
| Neg_kmetabolic | 1/s | 0.0010 | 69788.0 | 0.0001 |
| Neg_kdelay2d | 1/s | 0.3000 | 406.71 | 0.0900 |
| Neg_kdelay2c | 1/s | 0.0500 | 0.1400 | 0.0001 |
