# Supplementary material for: Mechanistic model for human brain metabolism and its connection to the neurovascular coupling
Source: PLoS Comput Biol. 2022 Dec 22;18(12):e1010798. doi: 10.1371/journal.pcbi.1010798 (PMC9822108; doi:10.1371/journal.pcbi.1010798)
Supplement: Attachment — Submitted filename: ReviewerResponse.docx [file pcbi.1010798.s008.docx]

Our replies to the reviewers comments are presented point-by-point below: reviewer comments are in **bold font,** and our responses in non-bold font.

**Reviewer #1: The authors integrated a simple computational model of brain energy metabolism into previously published models of neurovascular coupling.

My general consideration is that the fact that a model can fit some experimental data is not enough. Being able to get a good fitting of the data using a parameter optimization procedure is not a surprise, because model parameters are always many more than experimental measurements (i.e., the system is under-determined). What is important is to compare the optimized parameters with experimental equivalents.**

REPLY: This is a central aspect of our manuscript and modelling approach, which it is important to sort out. We do agree with Reviewer 1 that it is not an impressive or an important feature that a model can be made to agree with data, if optimized to those data. This is especially the case if highly flexible model structures are used, such as polynomials, and statistical and purely data driven models (e.g. ARX and ARMAX models, which has no physiological interpretation whatsoever). For mechanistically based models, such as is used here, achieving a good fit to data is already less trivial, and in this work, we have had to try a wide variety of different model structures, before an acceptable agreement with all data has been obtained. This process of acceptable and rejected model structures in itself provides vital insights regarding the processes that are at play in the studied system. However, the most important aspect to consider regarding this topic is that we also use independent validation data. While a sufficiently flexible model structure can be made to fit any data, if fitted to those data, overly flexible model structures are usually bad at correctly predicting new data, which they have not been fitted to. In fact, the more flexible the model structure, beyond a certain point, the worse the agreement with new data tend to be. This inability of too flexible models to correctly predict new data is known as the bias-variance trade-off (Cedersund and Roll, 2009; Cedersund, 2012). In summary, we therefore agree with Reviewer 1 that it is important to compare a model with experimental equivalents, and this is what we do when we compare the model to independent validation data (Figs 4, 5 & 6B).

We have added new text about this, and similar aspects concerning the modelling (mentioned below), in both the updated Introduction and the updated Discussion (all changes to the text are highlighted).

**But in order to do this, we need that each process (and by extension, each parameter) has a clear physiological significance. In the present model, the correspondence between model equation and physiology is lacking.**

REPLY: We do believe that mechanistic models should be physiologically and biochemically interpretable, and that there are many important advantages with such mechanistically based models. For those aspects of a model that is compared with data, that interpretation is automatically fulfilled, since the interpretation of the data and the model is (intended to be) identical. We also strive to construct a model that is physiologically and biochemically interpretable in all of its processes. However, we do undertake a minimal modelling approach, which means that the model is formulated in an as simple way as possible, while still being i) interpretable, and ii) able to agree with both estimation and independent validation data. We return to further details regarding the properties (strengths and weaknesses) of our minimal modelling approach in several of the other replies below.

Again: we have added more text about our approach to modelling, and regarding the minimal modelling, in the Introduction and Discussion (all changes highlighted).

**For instance, the authors aim to fit experimental 1H-MRS data for brain aspartate and glutamate concentration changes observed in response to sensory stimulation. These experimental findings have been interpreted by current literature as reflecting a complex dynamics of aspartate and glutamate across mitochondria within the so-called malate-aspartate shuttle (MAS), which includes (at least) two transaminations and two carrier-mediated co-transport processes. Well, in this paper there is nothing about the MAS, which implies that any parameter that results from the optimization procedure cannot be interpreted using current knowledge.**

REPLY: This is another important remark, since it highlights one of the fundamental and important differences there are between our minimal modelling approach, and those more detailed modelling approaches that Reviewer 1 argues for here, and below. It is true that the MAS is one of the mechanistic hypotheses for human brain metabolism that has been mentioned in previous literature, and in several of the previously developed models for human brain metabolism. However, how important MAS is for explaining the processes that we see here is not yet established. It might e.g. i) be a dominant process that dominates all of the concerned processes that produce what we see, ii) be a sufficient mechanism to alone explain the processes and dynamics we observe; iii) be a necessary process, without which you cannot understand or reproduce what you see; iv) be equilibrating so fast that it is negligible and can be assumed to be infinitely fast and in quasi-equilibrium with other slower, dominating processes, v) be so slow that it is unimportant on these time-scales and can be assumed constant, vi) be the bottle-neck process which dominates the dynamics and fluxes, in the sense that the others are much faster, vii) be unimportant for the dynamics that we observe, viii) be one of many alternatives explanations between which we cannot yet distinguish, etc etc. To answer any of these questions, or provide arguments for or against any of these different options, it is not enough to just include it in a model, based on previous models or stated hypothesis, or based on data from other systems (e.g. in vitro measurements). The answer to all of these points may differ from situation to situation, population to population etc. One must therefore analyse data from the intact system to be studied, and draw as much as conclusions as possible. Our results show that we can formulate a model that does not include the MAS mechanisms, and still produce all of the dynamics seen in the data. This means that we can conclude some things regarding the MAS mechanisms. We can conclude that they are not a necessary process to explain what we see in these data. This result in itself means that we can reject the case iii) above, and this might be because of iv), v), or viii), but we cannot say because of which. These are results and conclusions that we did not know before our modelling was done. Note that our results anyway do not exclude that MAS happens, or that it after all may be dominating, important, etc; alternative modelling – and probably also additional data – would be needed to draw or reject such conclusions.

**Let's make a simple example. The authors consider a generic glutamate-oxalacetate transaminase (GOT) maximal reaction rate (kmax-OAA in Eqs. 8 and 10; or Vmax_OAA1 in Supplementary Table 2) with an optimized value of 0.0007 s-1. How we compare this value (which is a result) with current literature? The present model lacks compartments, so we don't know whether it is the cytosolic or the mitochondrial reaction. The modeled reaction (OAA -> ASP) doesn't even include the full stoichiometry (OAA + GLU <-> OG + ASP), but this is (wrongly) splitted in two, with the other term (OG -> GLU) having a maximal rate (kmaxOG2in Eqs. 9 and 11; Vmax_OG2 in Table S2) of 0.0184 s-1. Experimental values for isolated cerebral mitochondrial GOT (Ferrari et al, J Neurochem 2018) are around 0.4 umol/min/mgprotein, which should roughly be 0.006 s-1 (assuming an aspartate concentration of 1 mmol/L and 1 mgprotein/ml in mitochondria). This number is substantially different from both the above-mentioned optimized values used in this model.**

REPLY: These detailed numbers and specific examples again make it clear what our results and approach show, and how the results should – and should not – be interpreted. If we would have included a multi-compartment model, we would have had more parameters, which would not have been necessary, in order to explain (and correctly predict) the currently studied data. Therefore, those additional parameters would have been more or less completely non-identifiable from data. In other words, we could not have determined values for those parameters. We illustrate this point in some additional analysis, which is included below, and in a new section of the supplementary text of the paper. As can be seen in the figure below, the expanded model’s parameters are non-determinable by many orders of magnitude. We also want to point out that also our model’s parameters are non-determinable by quite a big degree, which argues that our model could, and possibly even should, be simplified even further, especially if one is interested in the values of specific parameters. For this reason, to say that we have optimized them to a specific number (e.g. 0.0007, as Reviewer 1 argues above), is not a correct understanding of our results.

Furthermore, we are also quite cautious with comparing with – and especially using – data and parameter values that have been estimated previously, in previous models or in *in vitro* experiments. The corresponding values *in vivo* will differ from the measured *in vitro* values, and there is no way of knowing by how much they differ, without estimating the parameters to *in vivo* data, as we do here. Therefore, using *in vitro* values implies the introduction of an error, that you have no way of knowing how big it is, and that is not a satisfactory situation.

Finally, all model parameters in biological models are dependent on both the exact experimental situation (experimental protocol, cohort, etc; the parameters would even vary from experiment to experiment using the same cohort and protocol), and also on the model (if you formulate the model differently, the simulations and estimated parameter values will generally be different). Therefore, while it is not non-interesting to compare parameter values between models and different situations, great care needs to be taken, if one should not draw the wrong conclusions from such a comparison.

Again, we have expanded the introduction and discussion to highlight and discuss some of these issues and aspects of our results and approach more extensively.

**For these reasons, in this particular review, I did not examine the Results and the Discussion sections, because I have quite some concerns on the modeling approach. Once these are solved, I'll be happy to provide further feedback.**

REPLY: We have done our best to respond to these different concerns, by both new text and by new modelling results, as is hopefully clear both above and below.

**Main concerns

- The very premise (abstract and introduction) that there is a lack of mathematical models integrating vascular and metabolic responses to stimulation is not entirely correct. There were published papers well before the first one published by the authors (Lundengård et al., PLoS Comput Biol 2016) that attempted to do exactly that (although with different degree of accuracy in mechanistic details and possibly different scientific questions), including but not limited to De Pitta et al., J Biol Phys 2009, Mangia et al., J Neurochem 2009, and DiNuzzo et al. J Neurophysiol 2011. I guess there is much more.**

REPLY: Reviewer 1 is correct that there exists prior published works that aim to explain the connection between vascular and metabolic responses to neuronal stimulation. Furthermore, as the reviewer mentions, these prior works vary in their degree of mechanistic detail. It was never our intention to suggest that there are no prior works that have integrated both the vascular and metabolic responses. Rather, we aimed to show that the currently most mechanistically detailed description of the neurovascular coupling (Sten et al. 2020, 2021) lacks a detailed description of the metabolic response to neuronal stimulus. Furthermore, the existing works that have connected the vascular and metabolic responses have mainly done so using the Balloon model (Buxton et al. 1998) which is mainly a phenomenological model that lacks detailed mechanistic interpretations. As such the purpose of the current work is to develop a *minimal* model for the cerebral metabolic response that could explain the selected literature data, and to connect such a model to the detailed mechanistic description of the NVC presented in Sten et al. 2017. Finally, we also cannot find a previous metabolic model that describes our selected metabolic MRS-data, which means that these data do not come with a model-based data analysis. For all these reasons, we still believe that there is a need for the analysis and modelling done in our new manuscript.

To address and clarify these issues, we have revised the manuscript and expanded the reference list in line with the comments of the reviewer, and the reasoning above, such that the premise of the manuscript is hopefully more clearly conveyed (Introduction, 3^rd^ paragraph).

**- Contrary to already published models of brain activity/metabolism/blood flow, the present model does not include compartmentalized energy metabolism (e.g., neurons and astrocytes). Neither it includes glycogen metabolism. Neither it includes the main enzyme-catalyzed reactions (e.g., glycolysis). Neither it includes the main transport reactions (e.g., glucose and lactate transport across the blood-brain barrier). Many model rate equations are based on simple mass-action kinetics, which is wrong for many reaction/transport processes. For example, glucose transport (equation 4) in the brain is described by reversible Michaelis-Menten kinetics that has well-established parameters (see Simpson et al., JCBFM 2007). Besides, several reactions are lumped together (e.g., glycolysis), which doesn’t allow to account for: (i) redox balance, (ii) allosteric modulation (e.g., PFK), and (iii) product-inhibition (e.g., HK). Why did the authors not use prior knowledge?**

REPLY: Reviewer 1 raises the concern that there are several aspects of the cerebral metabolism, such as compartmentalisation, glycogen metabolism, transport reactions and more, that are not considered in the presented model. These are valid points that warrants some discussion as to why we have not included these aspects in our model, and we have done some further analysis and rewriting to illustrate these aspects (these points are also already discussed above).

The model presented in the current work aims to be a minimal representation of the cerebral metabolism that can still explain the experimental training data (taken from Lin et al. 2012). The idea to create a minimal model is that we want a model that is on the same level of complexity as the data that is used to train the model.

The main problem with expanding the model to include further complexity is that we would create more parameter unidentifiability. In other words, if we would expand the model to introduce some of the mechanisms you suggest above, this would introduce mechanisms and parameters that can compensate for each other, and thus not be uniquely determined from data, but can behave in any way, as long as the other mechanisms matches that behavior. The only thing that is constant and required in order to achieve an agreement with the data is thus a joint property regarding the smaller processes. A minimal model tries to capture those joint properties directly.

To illustrate this problem with unidentifiability, we have now created a two-compartment version of the model presented in this manuscript (Fig S1, also shown below). This two-compartment model is fitted to the experimental data for the metabolic response (section 2.5.1) and the parameter distributions are determined in the manner described in sections 2.4.1 and 2.4.2 in the manuscript. If we compare the estimated distributions of four parameters (${k_{max}}_{pyr2}, K_{M_{Pyr2}}, k_{max_{Glut1}}, k_{max_{Glut2}}$), it becomes apparent that the parameter distributions of the single compartment model (Fig S2 blue, also shown below) are more well determined than the corresponding parameter distributions of the two-compartment model (Fig S2 red and yellow, also shown below). Figure S2 shows a comparison of these parameter distributions in the form of a box chart. In this figure, the distributions are plotted on the y-axis with the different parameters considered on the x-axis. The blue boxes represent the parameter distributions for the single compartment model, the red boxes show the distributions for the corresponding parameters in the neuron compartment of the two-compartment model, and the yellow boxes show the distributions for the corresponding parameters in the astrocyte compartment of the two -compartment model. As can be seen, the red and yellow distributions of the two-compartment model are several orders of magnitude wider than the corresponding distributions of the single compartment model (note that the y-axis is plotted in log-scale). This problem appears since the parameters in the original single compartment model represent a combination of the parameters in the two-compartment model. It should be noted that this problem of identifiability is the case for a number of parameters in the two-compartment model, including but not limited to the four parameters showcased in the example.

This example shows that adding additional complexity to the model in the form of multiple compartments i) does not improve the model’s ability to explain the data we have considered in the manuscript, but ii) that it does reduce the parameter identifiability. The same problem would likely be the case if we added e.g., glycogen metabolism, or expanded the metabolic pathways that are currently lumped together into single reaction.

Note that these results and remarks do not mean that these additional processes do not occur in the brain, or that we claim that they do not occur. There are several works that point to the importance of these processes for the cerebral metabolism. However, given the experimental data considered in this work, it is not possible to model these mechanisms and preserve well-determined parameters. To achieve well determined parameters for a more comprehensive model, we would need to consider additional data that could be used to limit the parameter solution space. This could of course be done, but there is a limit to how much published data is relevant to consider, and there is a clear limit on what is currently available, measured on real human subjects. We think that the amount of data we have presented in the manuscript is relevant for the current aims of this work. Having said this, considering additional data from other sources to expand the model is something that would be highly interesting in future works.

Finally, all models of biological systems are simplifications to some degree and, in our modelling approach, the available experimental data is the factor that determines the degree of simplification. In other words, even if we would add the mechanisms that are suggested by the reviewer above (glycogen storage, compartments, etc), the resulting model would still neglect the majority of existing knowledge that exists about these systems (e.g. that there is a wide range of different cell types, many more than two; that there are many more reactions that happens that are still assumed negligible or not important to include as independent reactions, etc). Thus, these additions would add more knowledge, but would a) remove us from presenting a minimal model, with fairly identifiable parameters and well-determined predictions, b) still be possible to expand in the same way to add further known mechanisms. We therefore think that it is more clearcut to present a minimal model, since this has the added benefit of improved identifiability of parameters and predictions.

Based on the reviewer’s comments we, do, however, realize that this aspect and trade-off in the design of the modelling had not been discussed and highlighted in the text. We have therefore expanded the discussion section to put further emphasis on the aspects explored in this answer. Also, the analysis regarding identifiability in response to different model choices have been included in the updated version of the manuscript and in the supplementary.

$$V_{PC}=\frac{k_{{max}_{pyr2}}*Pyr}{K_{M_{Pyr2}}+Pyr}, V_{PAG}=Glut*k_{max_{Glut1}}, V_{clear3}=Glut*k_{max_{Glut2}}$$

**- Last but not least, I am not fully convinced of the iterative approach used to validate the model (Section 2.2, Page 3). Specifically, even in the circumstance that some experimental data cannot be fitted using a specific reductionistic model, it doesn’t mean that this model is necessarily wrong and has to be discarded. Indeed, the a’dition of new components might introduce nonlinear behaviors that can explain observations. Do the authors have any reference to a methodology paper that implements such a model construction strategy and rigorously examine the consequences?**

REPLY: The modelling workflow that we have used in the present work is based on the general principle of hypothesis testing, which lies at the heart of modern science: if a model cannot explain data, it should be rejected, and otherwise tentatively accepted, and used to design new experiments, which potentially could refute the model. This has been the basis of science for centuries (since Galilei), and formalized and made popular in the works of e.g. Karl Popper. Regarding applications within systems biology, we have adopted the theory from system identification presented by e.g., prof. Lennart Ljung (Ljung, 1999), to the specific context of systems biology in e.g (Cedersund, 2006, 2012; Cedersund and Roll, 2009). This has been applied by us and others in a multitude of different papers (Swameye *et al.*, 2003; Müller *et al.*, 2004; Maiwald *et al.*, 2016, and most of our previous ~50 publications).

Shortly, some of the most important consequences of a minimal, hypothesis-centric, modelling approach are: i) a key conclusion is the *rejection* of a model, i.e. that a certain set of mechanisms are insufficient to explain data, which means that *something is missing*, ii) identifiable parameters, i.e. determination of parameter values from data; iii) well-determined predictions, sometimes called core-predictions, which – just as parameters – can be uniquely determined from data; iv) parameters and states are often lumped, but still mechanistically interpretable, i.e. we can e.g. describe the sum total of the fluxes through the reactions in glycolysis, but not all individual reactions; iv) you ignore regulations and mechanisms that are not needed to explain the data. In other words, the ignored mechanisms are either not active, or cannot be uniquely seen in the current data In our model, we e.g. do not need independent regulation of PFK, substrate inhibition of GK, etc to explain these data (same comment is made about the MAS hypothesis, mentioned in an earlier comment above). This does not mean that these processes and regulation do not happen, but it means that we cannot say anything about them, in the given experimental conditions.

A specific reply to this: “**Specifically, even in the circumstance that some experimental data cannot be fitted using a specific reductionistic model, it doesn’t mean that this model is necessarily wrong and has to be discarded. Indeed, the addition of new components might introduce nonlinear behaviors that can explain observations**.” REPLY: if the model cannot behave like the data, some critical component is missing, and needs to be added to the model. The expanded model, with the new added component, is then another model (mechanistic hypothesis), which is not rejected. The first model/hypothesis, on the other hand, is rejected. In this way, you can e.g. determine which components that are *needed* to explain certain data, i.e. that seem to be active in a certain experimental setting.

**Few other specific points**	**- Page 1 Line 12 (abstract): Strictly speaking the neurovascular coupling connects cerebral activity and blood flow, while neurometabolic coupling connect cerebral activity and glucose/oxygen consumption. The same applies to Page 2 Lines 37-38 (introduction).**

REPLY: The manuscript has been revised to clarify the distinction between the neurovascular and neurometabolic couplings.  **- Page 2 Line 32 (introduction): I would include examples (e.g., glucose in the form of glycogen, and oxygen in globins). Yet, while oxygen storage is very limited, glucose storage as glycogen is quite substantial (Oz et al, Neurochem Res 2017).**

REPLY: The manuscript has been revised to add this reference, and the corresponding sentence has been adjusted accordingly.  **- Page 4 Line 115 (Section 2.3.1): Why it has to be a visual stimulation? It doesn’t seem that there is any particular detail in the stimulation equations 2 and 3 that is specific for the visual system, isn’t it? It could well be a cognitive stimulation, too. Please justify or remove “visual”. The same applies to the Results section. How is this model specific for the visual cortex?**

REPLY: The reviewer is correct in that the stimulation does mathematically not have to be specifically a visual stimulation. However, the model has been developed based on data from visual stimulations of the visual cortex, and is thus primarily a model for the visual cortex. Therefore, the most appropriate usage of the model is for visual stimulations. The manuscript has been revised to clarify this aspect.
 **- Page 4 Eq.3: Why the decay is soft (exponential), while the onset is not? Normally, in the literature stimulation functions consisting of a constant term plus a gamma function are used.**

REPLY: We have added a reference to other papers that use this alternative way of describing the onset. This choice is used by us for simplicity, i.e. because we undertake a minimal modelling approach (see above).	**- When using Michaelis-Menten kinetics, I suggest referring to maximal reaction rate as Vmax (this is the standard), while k is commonly used for mass-action kinetics.**

REPLY: We are aware of the standard convention to refer to the maximal reaction rate constant as Vmax. However, in our case we think that naming these maximal reaction rate constants as Vmax might cause confusion, since we call the reaction rates V, and different reactions rates Vx. Therefore, we opt to keep the notation as it is. If the reviewer is insistent on this, we can of course change things around even more, and also change the notations of the fluxes. However, there is always a risk that errors are introduced when changing notations at a late stage.	**- Page 5 equation 5: Please use “blood” not “body” for subscript to glucose.**

REPLY: The subscript “body” has been replaced by the subscript “blood“ in the term $Glucose_{Body}$, in equation 5, in accordance with the reviewer’s suggestion.

**- Page 5 equation 6: The reaction rate equation VTCA1 is conceptually wrong (multiplication of two MM equations). By the way, the whole process (VTCA1-VTCA2) doesn’t take into account carbon dioxide (so some mass is missing).**

REPLY: The expression used in equation 6 is implemented in the given from to give the expression a saturation with respect to both pyruvate oxaloacetic acid simultaneously. This is a standard way of formulating such dual regulations, with the arguably most simple way of assuming cross-talk between these regulations. However, the rate expression is not to be interpreted as two Michaelis-Menten expressions multiplied together, in the sense that the expression is not derived from elementary reactions. Instead, the chosen rate expressions are minimal descriptions of regulations that are needed to explain the data. For the same reason, we also discard the CO2 component; it is also not needed to explain the data.	**- Page 5 equation 7: I suggest replacing “lactate degradation” (ambiguous and possibly wrong) with “lactate clearance” (i.e., transport out of the brain tissue via blood or lymphatics), which is a physiological mechanism.**

REPLY: The term “lactate degradation” has been replaced by “lactate clearance”, in accordance with the reviewer’s suggestion.	**- Page 6 equation 10: What physiological process is responsible for “degradation” of aspartate?**

REPLY: Similar to the expression for Lactate, the term $V_{deg2}$ in equation 10 is used to allow aspartate to be further metabolised outside the current system and does not necessarily represent a single, specific physiological process, but the sum total of all processes that imply this result. The term $V_{deg2}$ has been renamed $V_{clear2}$ to clarify that the term represents the clearance of aspartate from the system.	**- Page 7 Line 197: GLUT is commonly used for “glucose transporter”. I suggest using PAG for glutaminase (i.e., phosphate-activated glutaminase).**

REPLY: The acronym GLUT has been replaced by PAG in the rate expression $V_{GLUT}$, in equations 11 and 12, in accordance with the reviewer’s suggestion.  **- Supplementary Table 2: please check out for optimized values too close to boundaries (e.g., ky2). By the way, please use the same identifiers (only one for each process) in the main manuscript text and in the Table.**

REPLY: The values presented in supplementary table S2 are the optimised parameter values, as well as the maximum and minimum values for each parameter that were found during the MCMC-sampling. Thus, the values in the table does not represent the boundaries for the optimisation algorithm but rather the boundaries to the range of acceptable parameter values obtained from the uncertainty analysis described in section 2.4.2. The S2 Table and the table legend have been revised to further clarify this.

The use of multiple identifiers for the parameter names have been revised such that the table matches the main manuscript text. We appreciate this clarification.  **- It would be nice to perform a sensitivity analysis after optimization.**

REPLY: This was done and described in section 2.4.2, and the results of the parameter sensitivities are presented in table S2. We understand that this might not be apparent for a reader and have therefore revised section 2.4.2 and table S2 to further clarify that this uncertainty analysis also encompasses the uncertainty of the parameter values.

-----

**Reviewer #2: Vibe authors propose an interesting model describing neurometabolic coupling. As any model, it has limitations, which are addressed by the authors in the manuscript. Many researchers a looking forward for such model to be easily available as predictor of brain metabolite changes in various experimental setups or neurological diseases or disease models. Having this model as an easy-to-use toolbox would be outstanding.**

**A few points require clarification: Blood compartment in the brain is not negligible, and blood glucose is about 5-fold larger than that in brain parenchyma. How is this brain and blood glucose concentrations handled when predicting MRS glucose ?**

REPLY: Reviewer 2 is absolutely correct in their assessment that the blood compartment contributes a substantial part of the total glucose in the brain. The amount of glucose in the local circulation is included in the model in the form of the model state $Glucose_{c}$, and this state directly determines the amount of glucose in the tissue (state $Glucose_{t}$). Thus, in the model glucose is split between a blood and a tissue compartment. Note, the experimental data consist of MRS measurements that cannot distinguish which compartment the measured glucose resides in. The current implementation of the model considers only the glucose levels in the tissue when fitting to the data. However, as the reviewer points out the amount of glucose in the circulation would be expected to be larger than the amount in the tissue. When comparing the model predicted glucose levels to the experimental data one might want to consider a combination of the two compartments but this was not done as the current implementation was able to adequately describe the data. However, since the reviewer has brought this issue to our attention, we have revised the discussion to elaborate further on this aspect of the current model implementation.	**As I can understand, brain glycogen metabolism is not considered in the model. This certainly contributes for glucose pools when brain activity is stimulated, and it is worth discussing.**
REPLY: As the reviewer states glycogen metabolism is not considered in the current model, and as elaborated further in the response to reviewer 1 above, this is because in this work we aimed to develop a minimal model of the cerebral metabolism and connect this model to existing models for the neurovascular coupling. However, the reviewer is correct that the effects of including glycogen metabolism in the model is worth discussing and we have therefore revised the discussion to accommodate this.

**Discussion on cmro2 and glycolysis: this paragraph needs some text clarification. Since glycolysis does not metabolize glycogen, there is quite some confusion in paragraph 2 of page 15.**

REPLY: We agree with the reviewer that the discussion regarding cmro2 and glycolysis could be clarified. We have thus revised this part of the discussion and in accordance with the previous answer also expanded the discussion to consider that glycogen metabolism is not currently considered in the model.

**The second limitation presented in the discussion is not really a limitation but a model design decision. thus, it is recommended that the authors elaborate on how including vascular regulation of flow and volume would impact model’s predictions. For example, could that improve BOLD signal rise prediction better? I am referring to the discrepancy in figure 6. Or could it predict better the levels of glucose, which have a substantial contribution from the vascular compartment?**

REPLY: Once again, we agree with the reviewer’s assessment that the discussion regarding the direct effects of the cerebral blood flow and cerebral blood volume could be elaborated further. We have therefore also revised this part of the discussion to also include how incorporating the vascular regulation could affect the model’s predictive capabilities.

**Reviewer #3: The paper uses the MEIGO software algorithm to investigate the addition of a metabolic model in an already published model of neuromuscular coupling. The authors show through the use of training data the time profiles of Lactate, Glutamate, Glucose and Aspartate following a visual stimulation of some 500 or more seconds. This stimulation is quite long and one has to wonder what other factors come into play during this time. These results it should be noted are relative changes rather than an actual baseline result.**

REPLY: These are correct and relevant comments. For these reasons, one has to be quite modest in expectations regarding what can be concluded from these quite limited data. Despite these limitations, these data constitute almost all available non-invasible data for human brain metabolism, and which thus warrant a sound and exhaustive analyzis, as well as a joint consideration together with other data, describing other aspects of the neurovascular coupling. Because of the limitations of available data, we undertake a minimal modelling approach, which seeks to construct a highly simplified metabolic network, which i) should provide some insights regarding the amount of reactions and regulations necessary to produce the data, ii) should be able to predict variables which are of the same nature as the data itself, but different in time-scale, intensity and stimulation patterns.

**Importantly the parameter list ( some 62 in all) as listed in Supplementary document S2 shows the “optimised” value as well as the upper and lower bounds determined during the global optimisation process.
This reviewer has to make a significant leap of faith in believing the algorithm of a global optimisation process in dimension 62 ( or above). In fact investigating the parameter list provides an argument which shows that the parameter values are either dependent on the training data or are incorrect. Looking at the parameter ky_{4}, its optimised value is 46.8257, but according to the algorithm the upper bound is 69990.4509 (still 4 decimal places ! really?) . Using equation (15) why should we believe the system is stable and of use to the physiological community. The parameter values may provide some result which matches the training data and thenceforth provide a result if other environmental changes occur but these dynamic steady states could reside anywhere in 62 dimensional space. If other parameters are perturbed by a small amount delta say will the dynamic steady state be moved by no more than a linear function of delta where that rate of change of function is small.**

REPLY: These are valid and central aspects, which we agree should be addressed.

Firstly, we want to address the seemingly high dimensionality of the parameters space (62 parameters). While this is a large number of parameters for the amount of data that has been used to train the model, it is should be noted that most of these parameters come from the previous neurovascular coupling model presented in Sten et al., NeuroImage, 2017. The model that describes the main addition in this manuscript – the metabolic response used to describe the MRS data – has only 18 parameters. This number was kept as low as possible, so that issues associated with large parameter spaces were minimized. In fact, we did this minimal approach to modelling in contrast to how most previous modelling of (human brain) metabolism has been done, which Reviewer 1 had some issues with. For this reason, we are glad that Reviewer 3 things that the model – if anything – is too big. This could imply that the current model strike a decent balance between physiological interpretability and representation of existing knowledge, and smallness in size compared to available data.

Secondly, regarding the 4 digits of the parameter values. This is a fair observation: these digits mean nothing, and have been removed. In fact, the uncertainties of many parameters are quite substantial, and we do not claim that any of the parameters are determined to a high precision, but each parameter can lie in a large region (sometimes uncertain by more than an order of magnitude). However, this does not mean that the individual parameters can be perturbed within these uncertainties at will. If one parameter is perturbed, other parameters also has to be changed accordingly, to still produce an acceptable agreement with data. The interesting aspect of the parameters are therefore captured by the totality of the many such acceptable parameter vectors. Each one of them means close to nothing, but what they say jointly, for e.g. a prediction, is a robust and well-determined prediction by the model (well-determined in the sense that the prediction has to lie within the plotted uncertainty range, in order for the model to be consistent with available data). When thusly considering the whole set of acceptable parameters, the model is thus robust to perturbations in parameters, and the model should mostly be used and analysed using the whole set (or using a represented sub-set) of acceptable parameters.

We have tried to make this more clear in the text about availability and usage of the model (in Materials and methods), and in the Discussion (all changes are highlighted).

**Secondly is the use of 10^5 samples sufficient (when using equation 17) to evaluate a data set given that the optimisation process occurs in 62 dimensions. Work from other groups suggest otherwise.**

REPLY: The reviewer is concerned that the number of samples used (10^5) might not be sufficient to adequately evaluate the available data. For this concern we would like to clarify that the 10^5 samples that are used for the MCMC-sampling are for estimating the parameter uncertainty distribution given the optimal parameter vector. The optimal parameter vector is obtained from the MEIGO toolbox’s enhanced scatter search (ess) algorithm, that has its maximum number of objective function evaluations set to 10^8. Both settings (10^5 samples for the MCMC sampling and 10^8 evaluations for the ess algorithm) are based on our previous model experience and have proven sufficient to correctly estimate an optimal solution for the model fitting and a reasonable parameter uncertainty. Furthermore, if we reproduce the results of the MCMC-sampling but increase the number of samples to 10^6, we see no substantial increase of the model’s parameter or simulation uncertainties. As can be seen in the figure below, where the parameter distributions resulting from 10^5 samples can be seen in blue and the parameter distributions resulting from 10^6 samples can be seen in orange. This confirms our previous experience that the MCMC sampling has converged.

**Also the equations set ( when looking at the parameter dimensions) are supposedly in dimensionless form , except time. Why was time not non-dimensionalised ?**

REPLY: The model was not non-dimensionalized *per se*, but rather formulated and developed using a minimal modelling approach. Since the data is relative data, describing only the change away from a previous steady-state, we see no reason to introduce state variables that are in the unit of concentration (say mM), and then introducing a measurement equation, with a new parameter that scales that concentration to the unitless measurement signal. This would only imply that more parameters are introduced (which - as is correctly pointed out by the reviewer above - should be kept as few as possible). Similarly, with such a model, the true values of metabolites, in a specific unit, would be arbitrarily uncertain. So the only thing we could say anything about is the relative changes from the steady states, which is what we now can show, when simulating the variables. This is the reason why the states (and therefore the corresponding rate constants) are described without the unit of concentration. In contrast, time is measured explicitly in the data, and therefore, we do not want to make time unitless.

We make this design choice more explicit in the description of the manuscript.

**Finally, there seems to be no information on the family of models that are not rejected by the optimisation process.**

REPLY: The family of models that were not rejected by the optimisation process are reported in the form of the uncertainty analysis described in Section 2.4 (and the uncertainty areas that are shown for the model predictions; they describe the total uncertainty for all non-rejected models). The resulting parameter uncertainties are reported in supplementary Table S2. However, we understand that it is not clear that the values reported in Table S2 are the boundaries of the parameter uncertainties i.e., the boundaries of the parameter values for which the model is not rejected. We have therefore revised the manuscript and Table S2 to clarify what is reported in Table S2.

**References**
Cedersund, G. (2006) *PhD-Thesis: Core-box modelling – theoretical contributions and applications to glucose homeostasis related systems*. PhD Thesis. Chalmers University Press, Gothenburg, Sweden.

Cedersund, G. (2012) ‘Conclusions via unique predictions obtained despite unidentifiability – new definitions and a general method’, *The FEBS Journal*, 279(18), pp. 3513–3527.

Cedersund, G. and Roll, J. (2009) ‘Systems biology: model based evaluation and comparison of potential explanations for given biological data’, *The FEBS Journal*, 276(4), pp. 903–922.

Ljung, L. (1999) *System Identification (2nd Ed.): Theory for the User*. Upper Saddle River, New Jersey 07458: Prentice Hall PTR.

Maiwald, T. *et al.* (2016) ‘Driving the Model to Its Limit: Profile Likelihood Based Model Reduction’, *PLOS ONE*, 11(9), p. e0162366.

Müller, T.G. *et al.* (2004) ‘Tests for cycling in a signalling pathway’, *Journal of the Royal Statistical Society: Series C (Applied Statistics)*, 53(4), pp. 557–568.

Swameye, I. *et al.* (2003) ‘Identification of nucleocytoplasmic cycling as a remote sensor in cellular signaling by databased modeling’, *Proceedings of the National Academy of Sciences*, 100(3), pp. 1028–1033.
