# Supplementary material for: Mechanistic model for human brain metabolism and its connection to the neurovascular coupling
Source: PLoS Comput Biol. 2022 Dec 22;18(12):e1010798. doi: 10.1371/journal.pcbi.1010798 (PMC9822108; doi:10.1371/journal.pcbi.1010798)
Supplement: Attachment — Submitted filename: ReviewerResponse.docx [file pcbi.1010798.s009.docx]

**Author reply:** We once again thank the reviewers for their diligent reviews and valuable feedback on our manuscript. We have herein replied to each of the reviewers’ remarks and concerns in a point-by-point fashion. Our replies are highlighted in yellow for easier readability.

**Reviewer #1**: Having a model with the "same level of complexity as the data" (authors' words) also means that the predictive power of the model is accordingly of the same level. What the physiology community cares about is the ability to make some predictions, especially about what we cannot measure.

**REPLY**: We agree with the reviewer’s statement that the predictive ability of the model is the key aspect of a model. We also agree that *“the predictive power of the model is accordingly of the same level”* as the training data. However, this does not mean that a minimal model cannot make interesting predictions. For instance, in the manuscript we present that the model can predict the metabolic response to very short visual stimulations (Fig 6). Measuring such a metabolic response is not possible since the signal-to-noise ratio would be too low leaving the signal undetectable. Further, these predictions of short stimulation metabolic response are done simultaneously with a prediction of the BOLD-response. This predicted BOLD-response is validated by experimental data which increases the confidence that the prediction for the metabolic response is also reasonable, even though the response cannot be accurately measured. This likelihood is of course also supported by the fact that the model can both describe and predict other types of metabolic data. This shows that we can do a prediction of a situation that cannot be experimentally measured.

It is no surprise that model identifiability is of no interest at all (except as an academic exercise) in modern machine learning. For instance, deep learning models have many millions of hyper-parameters, which is always much larger than the data used to train the model. But this doesn't matter, as long as the model behaves well in the test-set, because the value of the parameters is not important, as these do not have any physical meaning (so that you can have different orders, signs, etc., leading to the same results). Biological modeling is not machine learning, and I would expect that parameter values do have a meaning.

**REPLY**: We agree with the reviewer and have nothing to add to the statement that machine learning models can be at a level of complexity far beyond the data they are explaining, and that the parameter values in such model often completely lack any physiological interpretation. However, we would argue that this is not the case for the model we have presented in this manuscript. The parameter values in our model do have biological meaning; they e.g. explain the rate of the different reactions considered in the model. We grant that the minimal modelling approach result in that the parameter values might not be directly mappable to all known metabolic reactions. Rather, parameters such as $k_{{\max_{\mathrm{gluc}}}_{t}}$represent an aggregated average reaction rate through the glycolysis as a whole. However, since glycolysis is largely a linear pathway, such an average rate of glycolysis as a whole is still an interesting property. In other words, while this simplified interpretation might not be detailed to the level that the reviewer is used to, we do not agree with the implication that the parameter values in our model lack any biological interpretation.

Thus, if the authors' aim at providing a "minimal model" (their words) with clear parameter identifiability, this should be exactly the reason for having well-identified mechanistic details instead of approximate reaction rate equations (mass-action) and stoichiometries (see my previous example of the MAS), as it is in the present model.

**REPLY:** In response to this point, we would like to argue that mass action kinetics can be an approximation of Michaelis - Menten kinetics and if we are within a certain operating range this approximation will be good enough. If that is indeed the case, it is within this operating range, not possible to say anything meaningful about the $K_{M}$ and $V_{max}$- values, since the model is only operating in a small linear regime. We can in such situations thus only say something about the approximated corresponding kinetic rate $k_{i}$, concerning that specific system, investigated in those particular operating conditions. In other words, the nonlinearity and saturation are not seen (technically speaking: excited) enough in those conditions for the independent Michaelis Menten parameters to be seen; instead, the ratio between these two parameters is all that can be identified from data; and it is this ratio that we measure using our single kinetic rate constant. All this means that if the model can still explain and predict experimental data, which the presented model can, it is not wrong to approximate a MM-expression as a mass action expression; it is a warranted and justified simplification. If a simplification produces a well-determined prediction for the desired observed model property, the model is still useful.

All in all, it is not very difficult to fit the experimental time-courses of aspartate, glutamate, glucose, and lactate using a simple first-order linear model with 4 equations and 16 parameters. Unfortunately, such a minimal model would be useless, regardless of however narrow would be the parameter distributions.

**REPLY:** It might be true that a very simple linear model would be sufficient to explain the time course data we have considered in this study. However, such minimal linear model would have very simplified – if any – biological interpretations of the parameter values. If such a linear model could explain data, it would not per definition be useless solely on the account of being simple. We would argue that the usefulness of any given model depends on the aims of the user. The model we present in this manuscript provides a good description of the considered training data, can accurately describe validation data to which the model has not been trained, provides predictions for quantities that cannot be measured (Fig 6, and first response above), and provides, to our knowledge, a first connection between a mechanistically detained model of the neurovascular coupling and the neurometabolic response.

Anyway, maybe the authors' aim is to address model identifiability. If so, as a reviewer I would suggest that the authors' revise the manuscript in order to put model identifiability as the main purpose of the study.
1. First, the authors should distinguish between structural and practical model identifiability.
2. Then they should provide calculations of structural model identifiability for linear (or pseudo-linear), like the mass-action used in present model, and non-linear kinetics (e.g., Michaelis-Menten). To my knowledge, this has only been demonstrated using linearized models. Furthermore, it is commonly assumed that the system has a unique (as well as locally and asymptotically stable) steady state, but this is not necessarily true in biological networks.
3. Then they should provide evidence that previously published models are unidentifiable, in order to justify the development of the present model.
4. Finally, they should demonstrate that the present model is identifiable and, most importantly, gives some clear advantage in terms of explainability of neurophysiological and biochemical processes compared to previously published models.

**REPLY:** We thank the reviewer for their thorough proposal for an identifiability study and agree that a structural identifiability analysis would be a valuable addition for the model analysis presented in this manuscript. We have therefore provided such calculations in the revised supplementary material and amended the manuscript accordingly. However, we do feel that extending such an analysis to previously published models, while interesting, is outside the scope of this manuscript.

In summary, the authors' answer that they gave up on mechanistic knowledge because otherwise the model might be unidentifiable, cannot be accepted without a clear demonstration of such claim.

**REPLY:** We do not agree with the reviewer’s conclusion that we have given up on mechanistic knowledge just because the model we have presented provides a simplified description of the cerebral metabolism. Our model can simulate biologically interpretable metabolic pathways such as glycolysis and the tri-carboxylic acid cycle, although in a simplified manner. The reason for this simplified interpretation is that the considered data does not provide enough information for a more detailed description of the system to be identifiable.

Finally, let me give a brief example of what I mean. The sole addition of glycogen metabolism (in astrocytes) by DiNuzzo et al (2010b, PMID: 20827264) to their previously published model of brain energy metabolism (DiNuzzo et al, 2010a, PMID: 19888285) provided a biochemical explanation for why astrocytes reduce glucose uptake during functional activation (for neuronal use). This mechanistic explanation (brought about by an increased complexity of the model) formed the basis for the glucose sparing by glycogenolysis (GSG) mechanism, which has been recently shown to be able to excellently fit human (fluxomics) data from more than two decades of 13C-NMR experiments (Rothman et al, 2022, PMID: 34994222), among other neurophysiological explanations. The authors' argument that "there is a limit to how much published data is relevant to consider" (authors' words) is of course dependent on what's the question underlying the developed model.

**REPLY:** We thank the reviewer for this interesting example and have no objections to the final statement that the data to consider depends on the research question.

Neuron-astrocyte interactions are extremely important, as these compartments have distinct roles in neurotransmission, glutamate-glutamine cycle, and ionic movements, all aspects that are critical to brain function. Thus, it goes without saying that I am not entirely comfortable with the authors' answer that "This example shows that adding additional complexity to the model in the form of multiple compartments i) does not improve the model’s ability to explain the data we have considered in the manuscript, but ii) that it does reduce the parameter identifiability. The same problem would likely be the case if we added e.g., glycogen metabolism, or expanded the metabolic pathways that are currently lumped together into single reaction." (authors' words). Okay, adding complexity sometimes does not improve fit of the data or might even reduce parameter identifiability, so what? The physiology community is interested in neurophysiological insights, not mathematical modeling.

**REPLY:** We agree with the reviewer that neuron-astrocyte interactions are important for brain function. Although, we would argue that for an insight to be meaningful it must be identifiable. If we have an insight that is not identifiable it is not an insight but rather a suggestion, and whether or not an observed model property is identifiable will depend on data. For example, if we want to evaluate the glutamate-glutamine cycle, the current considered data would likely not be sufficient to gain any identifiable insights regarding this cycle. However, in this manuscript we have demonstrated that the data is sufficient to provide insights regarding the dynamics of the metabolic response in the visual cortex, since the uncertainty of these predictions are well-determined. For instance, we have shown that the presented model can be used to predict the metabolic response for very short visual stimulation, thus providing insights for an observed model property that is not experimentally determinable.

I understand that the authors insist on building a minimal model, and hence they take out everything that is not necessary to explain data. To me, this approach does not make any sense. I might well be wrong, and since my opinion could be now biased, it is in the interest of the authors and the Journal that I withdraw from reviewing this manuscript. I hope some of my comments were useful to improve the paper, and I apologize with the authors and with the Editor.

We again want to extend our thanks to Reviewer #1 for providing these comments. Exchanges like this are the corner stone of the scientific process, and different opinions must be allowed to be held up against each other. We also appreciate that Reviewer #1 agrees to bow out from further reviewing of the manuscript, to expediate the finalization of the publication process.

**Reviewer #2:** The authors have extended the manuscript to address my previous concerns.

**Reviewer #3:** In terms of the length of time for the training data the authors have unfortunately missed the point of the question. That is what happens to the parameter values if the further data becomes available but with a shorter ( or longer) time ?Would the parameters change and if so why, can the model explain this?

**REPLY:** We thank Reviewer #3 for this further clarification of the question. In short, nothing would happen to the parameter values with different stimulation lengths; we assume that the parameters are constant with respect to time. It is of course possible that a more accurate description of the true system could be obtained by allowing the parameters values to change during the course of the simulation, but such changes are not included in our model. As a support for this choice, we note that our model and assumption of constant parameters are enough to explain estimation data and to predict validation data. Since we use a minimalistic hypothesis testing approach we do not strive to make the model more complex than needed i.e., we do not wish to make the model parameter time variant if that is not necessary.

by taking the minimal modelling approach the only outcome of the model is a set of parameters that exist for a single data set.

**REPLY:** Here we would wish to clarify that the same set of parameter values are used for all training data and predictions presented in the manuscript. This means that the parameter values do not solely describe a single set of data, but rather the same parameter values obtained from training the model to the metabolic response in Figs 2 and 3 are used to predict the metabolic responses presented in Figs 4 and 5, and the BOLD-response in Fig 6B. Hence, we show that the parameter values obtained from the model training can be translated to insights regarding scenarios for which the model has not been trained.

For the issue of the large dimensionality of the system, the previous work referenced, does this have exactly the same data set for which parameter values ( 62-18) are evaluated? If not what proof can be shown that allows a dimensional reduction of this type?

**REPLY:** To clarify, the training data for the BOLD-response (Fig 3) is the exact same data set used to train the previous model presented in Sten *et al.*, 2017, NeuroImage. However, it is not apparent that the expansion of the Sten 2017 model, presented in this manuscript, still can explain the same data. Therefore, we retrained the expanded model to the same data set to show that the expanded model can still explain the data for the positive and negative BOLD-responses as shown in Fig 3. We understand that this might not have been entirely clear for the reader. We apologise for this and have revised the manuscript in accordance with our answer here to hopefully clarify this issue.

Given, as the authors admit that the parameter values can have a vast range ( "an order of magnitude") what confidence do we have in the model? Even if we take into account the values relative to baseline.

**REPLY:** This is a fair and relevant question. We argue that the confidence in the model is derived from the fact that the model accurately can describe validation data that the model has not been trained to, as seen in Figs 4, 5, and 6. This shows that the information gained in the model training is applicable to scenarios outside the immediate training region of observed model properties. Furthermore, we would like to stress that confidence in the model will depend on what the model should be used for. If the main purpose of the model is an accurate estimation of certain parameter values, then a well determined uncertainty for these parameter values is important. However, since we herein are interested to see if the model can describe the metabolic response, we are more concerned with the uncertainties of the predictions of this metabolic response. As presented in Figs 4, 5, and 6 the uncertainties of these predictions are well-determined. These well-determined predictions also demonstrates that even if we do have some unidentifiable parameters it does not necessarily translate to unidentifiability in all of the model’s predictions.

The authors say
"The interesting aspect of the parameters are therefore
captured by the totality of the many such acceptable parameter vectors. Each one of them
means close to nothing, but what they say jointly, for e.g. a prediction, is a robust and welldetermined
prediction by the model (well-determined in the sense that the prediction has
to lie within the plotted uncertainty range, in order for the model to be consistent with
available data)."
What does this mean ?

**REPLY:** We understand that this statement in our previous response might not be entirely clear and would like to clarify this statement. What we wanted to say is that when we are estimating the prediction uncertainties using the MCMC-sampling, the model prediction obtained from each individual set of parameter values provide little information on its own. But when all model predictions are considered together, we can conclude that the total uncertainty for the observed model property (in this case the predicted metabolic response for short-term stimulations), is well determined.

In terms of the use of 10^5 samples and the authors graph it must be noted that since the ordinate is a log scale variations that look small between 10^5 and 10^6 may not be small at all. Indeed some of the parameter variations are substantial.

**REPLY:** We agree with the reviewer that there are several parameters that have a substantial difference between the 10^5 and 10^6 samplings. We also agree that the logarithmic scale of the plot somewhat diminish these differences. However, we want to stress that the main point is not the value of individual parameters but rather the uncertainty of the predictions. Even for the 10^5 sampling the uncertainty of the predictions is small enough to still be useful as demonstrated in Figs 4, 5, and 6. Further, if we evaluate the differences in prediction uncertainties obtained from the 10^5 and 10^6 samplings respectively, we see that there are no major differences in the prediction uncertainties (see figure below). We apologise that this was not clear from our initial response to the reviewer’s concerns.

***Figure shows the predictions presented in Fig 6,*** *with an added comparison between sampling the uncertainty with 1e5 samples (blue shaded areas) and sampling with 1e6 samples (green shaded areas). Minor differences can be observed although the overall profile and magnitude of the uncertainties remains unchanged. Suggesting that the model uncertainties has converged for at 1e5 samples, at least for these predictions.*

 ***Figure shows the predictions presented in Figs 4 and 5,*** *with the added comparison between sampling the uncertainty with 1e5 samples (blue shaded areas) and sampling with 1e6 samples (green shaded areas). Minor differences can be observed although the overall profile and magnitude of the uncertainties remains unchanged. Suggesting that the model uncertainties has converged for at 1e5 samples, at least for these predictions.*
